# Complex Differential Diagnosis between Primary Breast Cancer and Breast Metastasis from EGFR-Mutated Lung Adenocarcinoma: Case Report and Literature Review

**DOI:** 10.3390/curroncol28050292

**Published:** 2021-08-31

**Authors:** Carmine Valenza, Francesca Maria Porta, Alessandra Rappa, Elena Guerini-Rocco, Giuseppe Viale, Massimo Barberis, Filippo de Marinis, Giuseppe Curigliano, Chiara Catania

**Affiliations:** 1 Division of New Drugs and Early Drug Development for Innovative Therapies, European Institute of Oncology, IRCCS, Via Ripamonti 435, 20141 Milan, Italy; carmine.valenza@ieo.it (C.V.); giuseppe.curigliano@ieo.it (G.C.); 2 Department of Oncology and Hemato-Oncology (DIPO), University of Milan, Via Festa del Perdono 7, 20122 Milan, Italy; elena.guerinirocco@ieo.it (E.G.-R.); giuseppe.viale@ieo.it (G.V.); 3 Division of Pathology and Laboratory Medicine, European Institute of Oncology, IRCCS, Via Ripamonti 435, 20141 Milan, Italy; francescamaria.porta@ieo.it (F.M.P.); alessandra.rappa@ieo.it (A.R.); massimo.barberis@ieo.it (M.B.); 4 School of Pathology, University of Milan, Via Festa del Perdono 7, 20122 Milan, Italy; 5 Division of Thoracic Oncology, European Institute of Oncology, IRCCS, Via Ripamonti 435, 20141 Milan, Italy; filippo.demarinis@ieo.it

**Keywords:** EGFR-mutated lung adenocarcinoma, breast metastasis, lung neoplasms

## Abstract

We present a case of a woman with epidermal growth factor receptor (EGFR)-mutated lung adenocarcinoma who received gefitinib for 2 years and obtained a partial response. The patient then developed liver metastasis and a breast lesion, displaying high estrogen receptor (ER) expression and harboring the same EGFR mutation. From the radiological studies, it was not possible to make a differential diagnosis between primary breast cancer and breast metastasis from lung cancer. After the removal of the breast nodule, thanks to the clinical history, radiology, and above all, molecular and immunohistochemical investigations, a diagnosis of breast metastasis from lung adenocarcinoma was made. This case emphasizes the importance of a comprehensive clinical, pathological, and molecular analysis in the differential diagnosis between primary breast cancer and metastases from extramammary tumor to guide adequate treatment decision making.

## 1. Introduction

Breast metastasis from extramammary malignancies are very rare, accounting for only 0.4–1.3% of all breast cancer cases [1,2]. Apart from contralateral breast cancer and hematological malignancies, the most common tumors that metastasize to the breast are malignant melanoma, lung cancer, renal cell carcinoma, and ovarian tumors [3].

Lung cancer is one of the most common sites of breast metastasis origin among carcinomas [4], representing 16–33% of all breast metastasis cases [5,6], and in international literature, about 250 cases have been described [4,7]. In these series, lung adenocarcinoma represents the most common histotype [8]. However, differential diagnosis between primary breast cancer and breast metastasis from lung adenocarcinoma remains challenging.

We reported an unusual case of metastasis to the breast from epidermal growth factor receptor (EGFR)-mutated and estrogen receptor (ER)-positive lung adenocarcinoma and provide a review of the literature focused on the breast as metastatic site from EGFR-mutated lung adenocarcinoma. Before writing this article, we studied and discussed the literature on PubMed with pathologists, in search of all the diagnostic and therapeutic pitfalls about the differential diagnosis between primary breast cancer and breast metastasis from lung adenocarcinoma.

## 2. Case Report

The patient is a 63-year-old with a height of 160 cm and a body mass index (BMI) 23.44. She is a Caucasian female without relevant comorbidities who has never smoked and who was diagnosed with a lung nodule in December 2015. The patient did not take any medication.

After a right upper lobectomy with lymphadenectomy, the histological diagnosis was pT2a pN1 stage IIB acinar-predominant lung adenocarcinoma.

In accordance with the disease stage, the patient received adjuvant treatment with cisplatin and vinorelbine, interrupted after one cycle because of acute renal failure and severe neutropenia.

In June 2017, as a consequence of positron emission tomography (PET) and endobronchial ultrasound transbronchial needle aspiration (EBUS-TBNA) detection of right hilar lymph node recurrence, the patient underwent ablative stereotactic radiotherapy at the level of hilar adenopathy (35 Gy/7 fractions). Molecular analyses were then performed on DNA/RNA extracted from a lung surgical resection specimen using a broad next-generation sequencing (NGS) panel (Oncomine Comprehensive Assay v3, Thermo Fisher Scientific, Waltham, MA, USA), and EGFR 19 deletion mutation (p.E746_A750del c.2236_2250del) was detected.

In July 2018, follow-up whole-body PET revealed disease progression with right pleural effusion intrascissural pleural nodules and right hilar lesion increase. Consequently, from September 2018 to October 2020 the patient received gefitinib (250 mg/die) with partial response.

In September 2020, whole-body computed tomography (CT) showed progressive disease with 19 mm liver lesion, a breast nodule, and a right hilar adenopathy.

Liquid biopsy was performed, but no T790M EGFR resistance mutation was detected. The patient underwent a tissue biopsy of the liver that showed an adenocarcinoma with positive thyroid transcription factor-1 (TTF-1) immunohistochemical staining, leading to the diagnosis of metastasis from lung adenocarcinoma. DNA extracted from biopsy specimen was subjected to NGS analysis (custom 26 panel, Thermo Fisher, Waltham, MA, USA), revealing the presence of the driver EGFR mutation (exon 19, p.E746_A750del c.2236_2250 del) and the EGFR resistance mutation (exon 20, p.T790M c.2369C>T).

Starting October 2020, the patient was given a second-line targeted therapy with osimertinib (80 mg/die). Meanwhile, the nodule in the right pectoral fascia was evaluated with mammography and ultrasound, which identified a 7 mm hypoechoic nodule, with partially undefined margins, without calcifications (Breast Imaging-Reporting and Data System (BI-RADS): 4a). After a fine-needle aspiration of the nodule showing TTF-1-positive and ER-positive tumor malignant cells, a quadrantectomy with sentinel lymph node biopsy was performed. Sentinel lymph node biopsy represents the gold standard for early breast cancer surgery and was necessary because of the uncertainty about the origin of the disease (primary breast cancer versus metastasis from lung cancer). This intervention made it possible to obtain much histological material to perform all the molecular and immunohistochemical analyses necessary to define the origin of the tumor.

Histologically, malignant tumor with glandular differentiation infiltrating the skeletal muscle tissue was seen. The neoplastic cells showed immunoreactivity to TTF-1, ER (95%), progesterone receptor (PR, 1%), human epidermal growth factor receptor 2 (Her-2/neu, 40%, ASCO/CAP score 2+), and Ki-67 (14%) and no expression of GATA-binding protein 3 (GATA-3). EGFR mutation (exon 19, p.E746_A750del c.2236_2250del) was detected with NGS molecular analysis.

Immunohistochemistry was retrospectively performed on archival lung and liver specimens. Neoplastic cells in the liver showed ER (90%) and Her-2/neu (40%, ASCO/CAP score 2+, FISH negative) expression. Positive ER (75%) and PR (2%) but negative Her-2/neu immunostaining were seen in the lung.

In December 2020, follow-up whole-body CT revealed liver partial response and hilar stable disease.

Therapy with osimertinib is still ongoing, and the woman has no evidence of tumor progression at the time of writing this report, and she has persistent, great clinical benefit.

### Pathological Features and Differential Diagnosis

**Figure 1:**
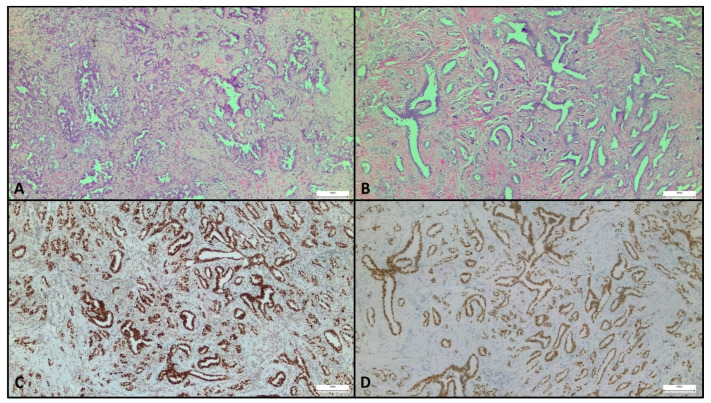
Representative micrographs of primary lung neoplasia and breast metastasis. Lung adenocarcinoma displayed a predominant acinar growth pattern (**A**). Breast tumor showed similar morphological appearance (**B**) and strong and diffuse expression of thyroid transcription factor-1 (TTF-1) (**C**) and estrogen receptor (ER) (**D**). Original magnification, 200×.

This patient originally presented with a typical lung adenocarcinoma, from both the morphological and the immunohistochemical points of view. It consisted of a moderately differentiated proliferation of cells growing in an acinar pattern, partially surrounded by desmoplastic stroma (Figure 1A).

The appearance of a mammary nodule after a 5-year interval, on the other hand, presented a more difficult diagnostic challenge from clinical and pathological points of view.

Fine-needle aspiration of the lesion identified the presence of malignant tumor cells positive for both TTF-1 and ER and negative for GATA-3, an immunophenotype compatible with the known lung malignancy but not specific enough to exclude the possibility of a primitive mammary neoplasm. Formalin-fixed paraffin-embedded histologic samples obtained after surgical excision of the nodule showed a morphology superimposable to that of the lung primary neoplasia (Figure 1B). However, this observation alone was not enough to exclude a mammary origin: primary NST breast cancer often presents as a proliferation of ductal structures, whose pattern can be similar to the acinar growth of a lung adenocarcinoma.

For this reason, an immunohistochemical panel was performed. The neoplastic proliferation was positive for TTF-1 (Figure 1C), ER (Figure 1D), PR, and HER2 but negative for GATA-3. The morphology and the immunophenotype, coupled with the patient’s clinical history, strongly favored lung rather than breast origin. The diagnosis of a breast recurrence of lung adenocarcinoma was confirmed by molecular analysis that revealed the presence of the same EGFR mutation detected in the primary lung neoplasia (exon 19, p.E746_A750del c.2236_2250del NM_005228.4).

## 3. Literature Review: Results and Discussion

For this review, a systematic literature search was performed on 24 April 2021 in Medline (PubMed); the keywords were: “metastatic breast cancer”, “lung cancer”, “differential diagnosis of breast metastasis”, “non small cell lung cancer”, and “EGFR-mutated lung cancer”. Articles about only breast cancer or lung cancer were excluded, and all case reports were included.

From 2000 to April 2021, 12 cases of breast metastases from EGFR-mutated lung adenocarcinoma were reported in the PubMed database, including our case; we summarized the clinical and molecular features of patients in the following table (Table 1).

Eleven patients (91%) were female, and the median age was 60.5 years; 8 patients (67%) never smoked. Six patients (50%) were diagnosed with stage IV lung adenocarcinoma from the beginning, but in most (91%) patients, the breast metastasis was metachronous [8]. However, despite the clinical history, the timing of metastasis onset is not diriment in differential diagnosis between primary and secondary breast cancer because of the high incidence of primary breast cancer among the female population.

In 8 patients (67%), the breast metastasis was hypothesized at physical examination, with the finding of a palpable mass (50%) or skin inflammation (50%). In fact, breast metastasis clinically appears as a superficial, firm, well-circumscribed, nontender, painless, and rapidly growing palpable mass with a predilection for the upper outer quadrant [6,19]; if it grows close to the skin, it can induce skin inflammation that mimics inflammatory breast cancer. Although skin and nipple retractions are uncommon [20,21], the physical examination is not specific.

In 11 patients (91%), the breast metastasis was ipsilateral to lung cancer with a statistically significant correlation between the lung cancer side and the breast metastasis side (*p* = 0.015). This evidence supports the Huang hypothesis of a lymphatic route that connects the parietal pleura to the breast, passing through the ipsilateral axillary lymph nodes [11]. However, in the cases of patients reported in the tables with ipsilateral breast metastasis, a statistically significant correlation between pleural effusion, axillary lymph node involvement, and breast ipsilateral metastases was not observed in Fisher’s exact test. Indeed, an extramammary tumor can metastasize to the breast through a lymphatic or a hematogenous route [22].

Imaging has low specificity in differential diagnosis between primary breast cancer and metastasis to the breast from extramammary cancer [6].

Differential diagnoses between primary breast cancer and extramammary metastasis in a morphological pattern is complicated, even if the pathologist knows the patient’s oncological history; about a third of lesions do not show specific histological features [23]: for example, poorly differentiated lung adenocarcinoma can show morphological features similar to triple-negative breast cancer [4]. Extramammary tumors are characterized by the absence of these histopathological signs: associated carcinoma in situ, desmoplastic reaction, elastosis, and microcalcifications; but even these clues are not 100% specific [23].

Immunohistochemistry is crucial for differential diagnosis between primary breast cancer and metastasis from lung adenocarcinoma. Given that there is no single marker with 100% sensitivity and specificity (Table 2), an immunohistochemical panel is often needed.

Both breast and lung cancer showed a CK7+/CK20− immunostaining profile [4]. Gross cystic disease fluid protein 15 (GCDFP-15) is expressed in about 60% of mammary carcinomas, but was also found in 5.2–15% of lung adenocarcinomas [24,25,26]. GATA-3 is expressed in about 67–95% of breast cancers and in up to 8% of lung adenocarcinomas [27]. Napsin A is expressed in 84% of primary lung adenocarcinomas, but it can be expressed in 14.6% of breast carcinomas with apocrine features [28,29]. TTF-1 is expressed in 70–80% of lung adenocarcinoma, but can also be found in 2.4–2.8% of breast cancers [30,31,32].

Finally, ER is expressed in 80% of mammary carcinomas [33] and in 7.6–27.2% of lung adenocarcinomas, depending on the antibody clones [34]. The progesterone receptor (PR) is expressed in 60% of breast tumors and in 1.6–54.8% of lung adenocarcinomas [35,36]. Two ER isoforms are described: alpha (ERα) and beta (ERβ). The latter is the predominant type of ER in NSCLC. On the other hand, the former seems to be restricted to specific subtypes of lung adenocarcinoma, such as those EGFR mutated [35]. In a retrospective series of resected NSCLC tumors, ERβ expression was detected more commonly in tumors from never smoker patients, especially in female patients [37].

In the series reported in Table 1, all breast metastases were positive for TTF-1, CK7, and napsin A and negative for GCDFP-15 and GATA-3. Only two cases showed ER expression, including the patient described in this report.

As far as molecular analysis is concerned, EGFR-activating mutations are observed in up to 30.3% of metastatic lung adenocarcinomas [38], especially in East Asian patients, never smokers, and women. Anyway, some authors reported these mutations (e.g., exon 19 deletion) also in 3–11% of Asian patients with triple-negative breast cancer [39,40,41]. In seven patients in the series presented in Table 1, the EGFR mutation was searched both in the lung and in the breast tissue to make the differential diagnosis, but the molecular analysis reached 100% specificity only in association with the clinical history.

In the cases collected in Table 3, breast metastasis treatment included surgery in two patients (16.6%), EGFR-tyrosine kinase inhibitor (TKI) in 9 patients (75%), chemotherapy in 5 patients (41.6%), immunotherapy in 1 patient (8.3%), and only best supportive care in 2 patients (16.6%), according to performance status (Table 3).

The role of breast metastasis local excision is controversial. Williams et al. demonstrated a better median overall survival (mOS; 15.5 vs. 8.1 months) in patients with metachronous breast metastasis who had surgery [41], but this population was heterogeneous and was not stratified according to the primary tumor site. In fact, in other studies the surgery does not lead to prolonged survival [42,43]. According to current knowledge, surgery should be considered only in an oligometastatic setting or in case of unclear differential diagnosis (with sentinel lymph node biopsy if a breast cancer is suspected).

Osimertinib represents the current first-line standard treatment of EGFR-mutated stage IV lung adenocarcinoma. In EGFR-mutated adenocarcinoma, it has been shown that estrogen can induce cell proliferation and tumor growth, also through EGFR signaling pathway interaction [44,45]. In fact, (1) 67% of EGFR-mutation-positive tumors exhibit a high expression of nuclear ERβ versus 37% in EGFR wild-type tumors [46], and (2) EGFR has been reported to directly phosphorylate ER at specific serine residues in 87.5% of ER-positive lung tumors [47].

In according to these data, preclinical evidence has shown that EGFR-TKI and antiestrogen have a synergistic effect on the proliferation inhibition of NSCLC cell lines [48]. Despite this evidence, two randomized phase II trials evaluating treatment with EGFR-TKI associated with antiestrogen in women with nonsquamous advanced-stage NSCLC were negative [49,50]. In the IFCT-1003 LADIE trial, the addition of fulvestrant to gefitinib did not improve progression-free survival both in EGFR-mutated and in EGFR-WT cohorts; furthermore, no PFS difference was observed regarding estrogen receptor alpha expression. In summary, despite in vitro evidence, no clinical trial supports the use of antiestrogens in ER-positive lung adenocarcinoma.

## 4. Conclusions

In conclusion, this clinical case underlines the importance of an in-depth diagnostic evaluation of women with breast lesion and a history of lung adenocarcinoma and the multidisciplinary management of these patients. Differential diagnosis between primary breast cancer and metastasis to the breast from lung adenocarcinoma is challenging and requires the combination of clinical, radiological, pathological, and molecular findings. This differential diagnosis is crucial for its therapeutic and prognostic value.

The main limitation of this article is the small number of clinical cases reported in the literature. Another limitation is represented by the clinical and biological heterogenicity of both breast and lung cancer; the choice of a precise histotype (adenocarcinoma) and a molecular subgroup (EGFR mutated) also aims to simplify this heterogeneous landscape.

Furthermore, in these cases of lung cancer expressing estrogen receptors, it would be interesting to evaluate both the activity of the association of the aromatase inhibitor or antiestrogen drugs with osimertinib and the addition of aromatase inhibitors/antiestrogens to osimertinib at progression during osimertinib treatment.

## Figures and Tables

**Table 1:** Case series.

Case (Author, Year)	Age, Sex	Smoke	Primary Lung Cancer	Initial Stage	PE before BM	BM Suspicion	Size BM	Metachronous (Time)	Ipslateral	EGFR Mut	IHC Marker	Axillary LN
Fukumoto 2011 [9]	65, W	N	Left Lower Lobe	IIIA	No	Imaging	Tiny nodule	6 years later	Yes	Ex 19 (L and B)	TTF-1+ ER- (B)	No
Sato 2012 [10]	57, W	N	Right Upper Lobe	IV	Yes	Clinical	Inflammatory breast tumor	12 months after	Yes	Ex 19 del (L) T790M (B)	CK7+ TTF-1+ ER-PgR- HER2- (B)	Yes
Huang 2013 [11]	70, W	NA	Left Upper Lobe	IV	Yes	Clinical	Fixed hard mass	3 months later	Yes	Ex 21 L858R mut (L)	TTF-1+ ER- PgR- GCDFP-15- (B)	Yes
Liam 2013 [12]	70, W	N	Right Lower Lobe	IV	Yes	Clinical	Inflammatory breast tumor	18 months later	Yes	Ex 20 ins (L)	TTF-1+ (L) TTF-1+ ER- PR- HER2- (B)	Yes
Jeong 2014 [13]	47, W	N	Left Upper lobe	IB	No	Imaging	1.3 cm	3 years later	Yes	Ex 19 del (c.2239_2247del9) (L and B)	ER- PR- HER2- GCDFP-15- ALK- TTF-1+ CK-7+ Napsin A+ (B)	No
Mirrielees 2014 [8]	58, W	F	Left Lung	IIIA	No	Clinical	1.3 cm	3 years later	Yes	Ex 19 non specified mut (L)	ER+ (20%) PR- HER2- TTF-1+ (B)	Yes
Dansin 2015 [14]	52, W	N	Left Upper Lobe	IV	Yes	Clinical	26 mm	Synchronous	Yes	Ex 19 del (L and B)	ER- PR- HER2- TTF1+ GATA-3- PAX8- (B)	Yes
Lee 2015 [15]	49, W	N	Right Upper Lobe	IIIA	Yes	Imaging	Inflammatory breast tumor	4 years later	Yes	NA (L)	CK7+ TTF-1+ CK20+ (L)	No
Lin 2016 [16]	49, M	C	Left Upper Lobe	IV	Yes	Clinical	5 cm	18 months later	No	Ex 21 L858R mut (L and B)	Chromogranin A+ Synaptophysin+ CD56+ TTF-1+ ER- GCDFP-15- HER2- (B)	No
Ninan 2016 [17]	67, W	NA	Right Lung	IIIB	NA	Clinical	Inflammatory breast tumor	NA	Yes	NA (L)	TTF-1+ CK7+ GATA-3- GCDFP-15- (B)	No
Ota 2018 [18]	69, W	N	Left Lower Lobe	IV	Yes	Clinical	Inflammatory breast tumor	12 months later	Yes	Ex 21 L858R mut (L and B)	ER- PR- HER2- (B)	Yes
Current case	63, W	N	Right Upper Lobe	IIB	Yes	Imaging	7 mm	5 years later	Yes	Exon 19 del (c.2236_2250del) (L and B)	TTF-1+ ER+ (75%) PR+ (2%) HER2- (L) TTF-1+ ER+ (95%) PR+ (1%), HER2 (2+), GATA-3- (B)	No

ALK: anaplastic lymphoma kinase; B: breast; BM: breast metastasis; C: current; CK7: cytokeratin 7; del: deletion; EGFR: epidermal growth factor receptor; ER: estrogen receptor; Ex: exon; F: former; GATA-3: GATA-binding protein 3; GCDFP-15: gross cystic disease fluid protein 15; HER2: human epidermal growth factor receptor 2; IHC: immunohistochemical; L: lung; LN: lymph node; M: man; mut: mutation; N: never; NA: not available; PAX8: paired box 8; PE: pleural effusion; PR: progesterone receptor; Syn: synchronous; TTF-1: thyroid transcription factor-1; W: woman.

**Table 2:** Immunohistochemical and molecular profile of lung adenocarcinoma and breast cancer.

IHC or Molecular Characteristic	Breast Cancer (Prevalence)	Lung Adenocarcinoma (Prevalence)
GCDFP-15	60%	5.2–15%
GATA-3	67–95%	8%
Napsin A	14.6%	84%
TTF-1	2.4–2.8%	70–80%
ER	80%	7.6–27.2%
PR	60%	1.6–54.8%
EGFR-activating mutations	3–11% (TNBC)	30.3%

EGFR: epidermal growth factor receptor; ER: estrogen receptors; GATA-3: GATA-binding protein 3; GCDFP-15: gross cystic disease fluid protein 15; IHC: immunohistochemical; PR: progesterone receptor; TNBC: triple-negative breast cancer; TTF-1: thyroid transcription factor-1.

**Table 3:** Breast surgery and Treatment.

Case	Breast Surgery	EGFR-TKI	CT	IT
Fukumoto 2011	Partial mastectomy	No	No	No
Sato 2012	No	No	CBDCA + PMX + Bevacizumab	No
Huang 2013	No	Erlotinib	No	No
Liam 2013	No	No	No	No
Jeong 2014	Lumpectomy and sentinel LN biopsy	Gefitinib	No	No
Mirrielees 2014	No	Erlotinib	CBDCA + PMX	No
Dansin 2015	No	Afatinib	5-FU + Epi + CTX	No
Lee 2015	No	Afatinib	No	Nivo + Ipi
Lin 2016	No	No	CDDP + VP16	No
Ninan 2016	No	No	No	No
Ota 2018	No	Erlotinib	CBDCA + Paclitaxel + Bevacizumab	No
Current case	Quadrantectomy	Osimertinib	No	No

5-FU: 5-fluorouracil; CBDCA: carboplatin; CDDP: cisplatin; CTX: cyclophosphamide; EGFR: epidermal growth factor receptor; Epi: epirubicin; ER: estrogen receptor; GATA-3: GATA-binding protein 3; GCDFP-15: gross cystic disease fluid protein 15; IHC: immunohistochemical; Ipi: ipilimumab; LN: lymph node; Nivo: nivolumab; PMX: pemetrexed; PR: progesterone receptor; TNBC: triple-negative breast cancer; TTF-1: thyroid transcription factor-1; VP16: etoposide.

## Data Availability

The data presented in this study are available in this article.
